# Readability of AI-Generated Patient Information on Glucagon-Like Peptide-1 Receptor Agonists

**DOI:** 10.2196/90572

**Published:** 2026-05-05

**Authors:** Tyler Williams, Ines Bilic-Curcic, Jonathan Hurley, Harisankeerth Mummareddy, Maja Cigrovski Berkovic, Silvija Canecki Varzic, Marina Gradiser

**Affiliations:** 1 Faculty of Medicine, Tulane University, New Oreleans, LA, United States; 2 School of Medicine, Tulane University, New Orelans, LA, United States; 3 University of Tennessee Health Science Center, Menphis, TN, United States; 4 Faculty of Kinesiology, University of Zagreb, Horvacanski zavoj 15, Zagreb, 10000, Croatia, 385 915128167; 5 Faculty of Dental Medicine and Health, University of Osijek, Osijek, County of Osijek-Baranja, Croatia; 6 Faculty of Medicine, University of Split, Split, Split-Dalmatia, Croatia

**Keywords:** health literacy, GLP-1RA, AI, readability, ChatGPT, Google Gemini, artificial intelligence

## Abstract

Artificial intelligence (AI)–generated content on glucagon-like peptide-1 receptor agonists (GLP-1RAs) gave informationally detailed responses, but its readability remains suboptimal for many patients. Incorporating literacy-sensitive design principles into AI health communication is essential to ensure equitable access to digital medical information.

## Introduction

Glucagon-like peptide-1 receptor agonists (GLP-1RAs) have become cornerstone therapies for type 2 diabetes mellitus and obesity, with additional benefits extending to cardiovascular risk reduction and metabolic health [1,2]. Their rapid clinical uptake has been paralleled by a surge in public interest and online information-seeking [3-5]. Artificial intelligence (AI)–based conversational tools are increasingly used by patients as informal sources of medical guidance. However, engagement with digital health information is strongly influenced by health literacy. Patient education materials are generally recommended to be written at or below an eighth-grade reading level to ensure comprehension across diverse populations [6,7]. Data from the OECD Survey of Adult Skills indicate that a substantial proportion of adults demonstrate limited literacy skills, raising concerns that complex digital health content may exacerbate existing health disparities [8]. Given the expanding role of AI-generated medical information, evaluating whether such content is accessible to patients is crucial. This study assessed the readability of AI-generated responses to common patient questions regarding GLP-1RAs, hypothesizing that language complexity would exceed recommended thresholds for patient-facing materials.

## Methods

A cross-sectional descriptive analysis was conducted using two large language models: ChatGPT (GPT-4.1; OpenAI) and Google Gemini (Gemini 2.5 Flash; Google DeepMind). Ten frequently asked patient questions related to GLP-1RAs were identified through review of online patient forums and routine clinical encounters. Topics included dosing, side effects, safety, insurance coverage, off-label use, and expected outcomes.

Each question was submitted to both models using identical prompts requesting a “clear, patient-friendly response.” Responses were collected verbatim between June and August 2025. All queries were submitted using the models’ web-based interfaces. Each query was entered in a new session to avoid retention of prior conversational context. No system-level prompts, role instructions, or parameter adjustments (eg, temperature, top_p) were applied; therefore, outputs reflect default model behavior as experienced by typical users. Identical prompt instructions were used for all queries across both platforms.

Readability was evaluated using three validated measures: (1) Flesch Reading Ease Score, where higher scores indicate easier readability; (2) Flesch-Kincaid Grade Level (FKGL), estimating required US school grade level; and (3) mean sentence length and word count as proxies for textual complexity.

Analyses were performed using ReadablePro software and cross-validated with Microsoft Word readability tools. In addition, two independent health communication experts qualitatively reviewed responses for tone, sentence structure, and technical language use. Paired *t* tests were used to compare mean readability scores between ChatGPT and Gemini responses. In addition to *P* values, paired mean differences with 95% CIs and effect sizes (Cohen *d_z_*) were calculated to assess the magnitude of differences.

Before readability analysis, all outputs were standardized to plain text format. Formatting elements such as bullet points, headings, and line breaks were removed to ensure consistent processing across readability tools.

The full list of patient questions, verbatim model outputs, and per-question readability metrics are provided in Multimedia Appendices 1-3.

## Results

Across all 10 questions, both models generated detailed responses ranging from 150 to 450 words. Gemini produced shorter sentences and more concise explanations, whereas ChatGPT responses were longer and more technically detailed.

**Figure 1.**
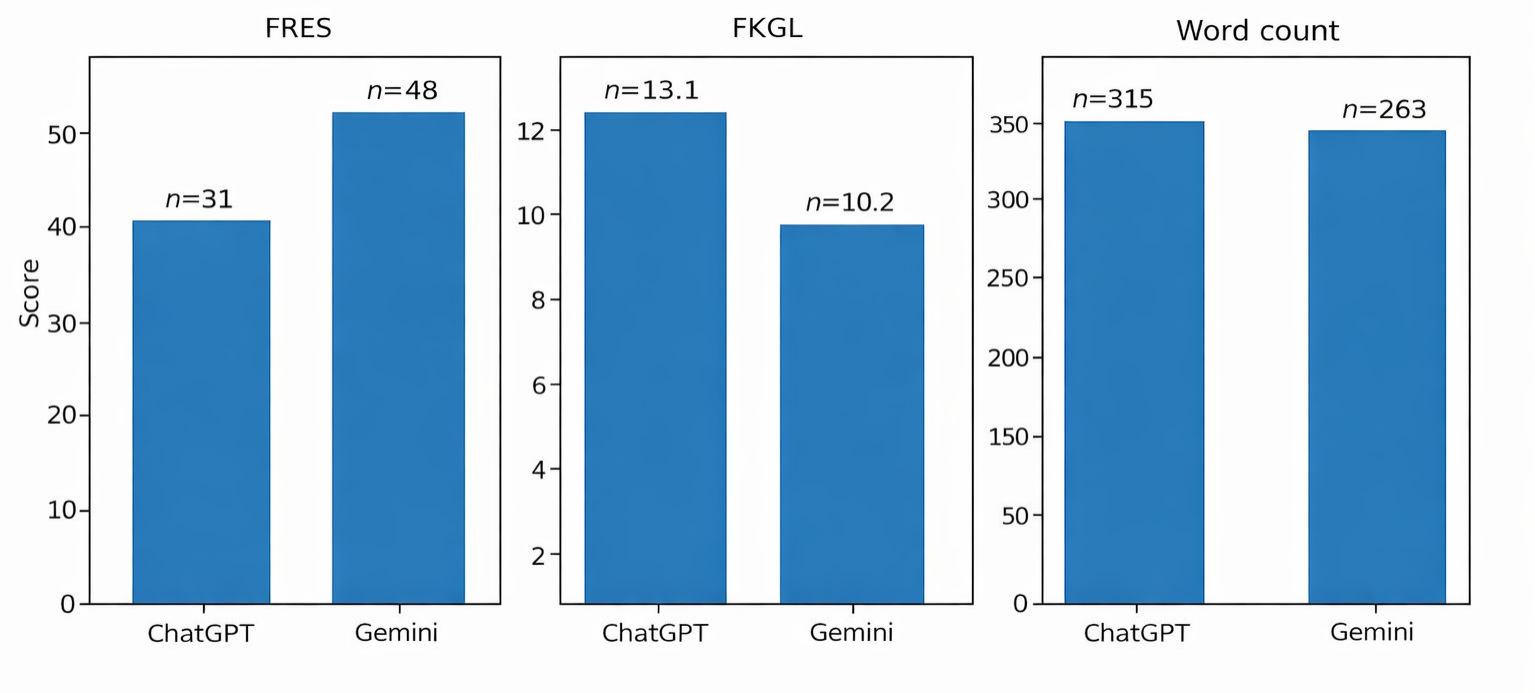
Comparison of readability metrics between ChatGPT and Google Gemini responses. ChatGPT responses demonstrated lower Flesch Reading Ease Scores (FRES) and higher Flesch-Kincaid Grade Levels (FKGLs) compared to Gemini, indicating greater linguistic complexity. Both models exceeded recommended readability thresholds for patient education materials.

Mean Flesch Reading Ease Score was significantly higher for Gemini than for ChatGPT (47.97 vs 31.65; *P*=.004), indicating superior readability (Figure 1). Similarly, mean FKGL was lower for Gemini (10.2) compared with ChatGPT (13.1), although both exceeded the recommended eighth-grade level.

Using FKGL as the operational measure, both models exceeded the recommended ≤8th-grade level for patient education materials by approximately 2‐5 grade levels on average.

Qualitative analysis revealed that ChatGPT frequently used complex sentence structures and specialized terminology, while Gemini responses adopted a more conversational tone with simpler vocabulary.

## Discussion

This study demonstrates that AI-generated patient information on GLP-1RAs varies significantly in readability depending on the model used. Although Gemini responses were more accessible than those generated by ChatGPT, neither consistently met recommended readability standards for patient education. These findings align with prior evidence showing that online health information frequently exceeds the literacy capacity of the general population [9-12]. These findings also highlight limitations of general-purpose AI models in health care contexts. Domain-specific, medically trained AI agents may provide more consistent, context-aware, and clinically appropriate outputs. Such systems could incorporate structured medical knowledge, terminology control, and longitudinal context, which are critical for reliable patient communication. In clinical applications, AI systems must also include mechanisms for uncertainty management and safety. When confidence in generated responses is low, fallback strategies, such as retrieval from validated medical sources or escalation to health care professionals, are essential to ensure reliability and patient safety.

Given that AI tools prioritize completeness and accuracy, readability may be unintentionally deprioritized. Without deliberate incorporation of plain-language principles, AI-generated health content risks widening disparities among individuals with limited literacy or digital health skills.

It is important to note that outputs from large language models are highly sensitive to prompting strategies. This study intentionally used identical prompts to simulate a real-world patient scenario, in which users typically enter similar queries across platforms without advanced prompt engineering. However, more sophisticated prompting approaches or orchestration layers may yield responses with substantially different readability profiles. Future studies should explore the impact of prompt design on health communication outcomes.

AI systems also offer the potential for interactive, bidirectional communication and personalization, which may enhance patient engagement. However, without appropriate attention to readability and comprehension, these advantages may not translate into meaningful improvements in patient outcomes.

An important opportunity for future development is the integration of literacy-adaptive mechanisms within AI systems. Tailoring responses to a user’s health literacy level, through simplified language or dynamic adjustment of complexity, could significantly enhance accessibility and patient understanding.

This study has several limitations. First, AI-generated outputs are subject to variability over time due to model updates and changes in system behavior; therefore, the findings reflect the specific models, settings, and time frame studied. Second, results may differ with alternative prompting strategies. Third, this study evaluated readability but did not assess factual accuracy or patient comprehension outcomes, which should be addressed in future research.

ChatGPT and Gemini provide clinically relevant responses on GLP-1RAs, but responses remain insufficiently readable for many patients. Integrating literacy-sensitive frameworks into AI health communication is essential to ensure equitable access to digital medical information and maximize the clinical benefits of emerging therapies.

Implementation of standardized readability frameworks within AI systems may represent a key step toward equitable digital health communication.

## Supplementary material

10.2196/90572 Multimedia Appendix 1 Patient questions used in the analysis.

10.2196/90572 Multimedia Appendix 2 Artificial intelligence–generated responses (verbatim).

10.2196/90572 Multimedia Appendix 3 Supplementary table.

## Abbreviations

AI: artificial intelligence
FKGL: Flesch-Kincaid Grade Level
GLP-1RA: glucagon-like peptide-1 receptor agonist
