# Supplementary material for: Readability of AI-Generated Patient Information on Glucagon-Like Peptide-1 Receptor Agonists
Source: JMIR Bioinform Biotechnol. 2026 May 5;7:e90572. doi: 10.2196/90572 (PMC13143154; doi:10.2196/90572)
Supplement: Multimedia Appendix 1 [file bioinform-v7-e90572-s001.docx]

Supplementary Appendix 1. Patient Questions Used in the Analysis

The following ten standardized patient-oriented questions were used as prompts for both AI systems:

1. What are GLP-1 receptor agonists, and how do they work?

2. What conditions are GLP-1 agonists used for?

3. What is the cost of GLP-1 agonists and is it covered by insurance?

4. Can GLP-1 agonists be used with other medications?

5. What are the common side effects of GLP-1 agonists?

6. Are GLP-1 agonists safe for everyone and for long-term use?

7. How much weight can you lose on GLP-1 medications?

8. How quickly do GLP-1 agonists work for weight loss and blood sugar control?

9. Can you stop GLP-1 medications once you’ve lost weight?

10. What’s the difference between Ozempic, Wegovy, and Rybelsus?
