# Supplementary material for: Readability of AI-Generated Patient Information on Glucagon-Like Peptide-1 Receptor Agonists
Source: JMIR Bioinform Biotechnol. 2026 May 5;7:e90572. doi: 10.2196/90572 (PMC13143154; doi:10.2196/90572)
Supplement: Multimedia Appendix 2 [file bioinform-v7-e90572-s002.docx]

Supplementary Appendix 2. AI-Generated Responses (Verbatim)

Q1. What are GLP-1 receptor agonists, and how do they work?

ChatGPT

GLP-1 receptor agonists (GLP-1 RAs) are a class of medications that mimic the action of glucagon-like peptide-1, a hormone naturally produced in the gut in response to eating. They are used primarily to treat type 2 diabetes and obesity.

They work by enhancing insulin secretion when blood glucose is elevated, suppressing glucagon release, delaying gastric emptying, and reducing appetite through central mechanisms. These combined effects improve glycaemic control and promote weight loss.

Google Gemini

GLP-1 receptor agonists (GLP-1 RAs), also known as incretin mimetics, are medications that mimic a natural hormone involved in regulating blood sugar and appetite.

They stimulate insulin release when blood sugar is high, suppress glucagon secretion, slow gastric emptying, and increase feelings of fullness. These effects help control blood glucose levels and support weight reduction.

Q2. What conditions are GLP-1 agonists used for?

ChatGPT

GLP-1 receptor agonists are primarily used to treat type 2 diabetes mellitus and obesity. They improve glycaemic control, promote weight loss, and may reduce cardiovascular risk in certain patient populations. Additional investigational uses include metabolic and liver-related conditions.

Google Gemini

GLP-1 agonists are mainly used to treat type 2 diabetes and obesity. They can also provide cardiovascular and metabolic benefits, including improved blood pressure, lipid levels, and reduced cardiovascular risk in selected patients.

Q3. Cost and insurance

ChatGPT

GLP-1 medications are expensive, typically ranging from $800 to $1,500 per month without insurance. Coverage is common for diabetes but less consistent for weight loss indications. Manufacturer programs may reduce costs.

Gemini

GLP-1 drugs often cost over $1,000 per month without insurance. Coverage varies widely depending on diagnosis, insurer, and plan type, with stricter criteria for obesity treatment.

Q4. Combination therapy

ChatGPT

GLP-1 agonists can be combined with medications such as metformin, SGLT2 inhibitors, and insulin. Careful monitoring is required due to hypoglycaemia risk with some combinations.

Gemini

GLP-1 agonists are commonly used alongside other medications, especially in diabetes. Interactions are generally manageable but require clinical supervision.

Q5. Side effects

ChatGPT

Common side effects include nausea, vomiting, diarrhoea, constipation, and abdominal discomfort. Rare complications include pancreatitis and gallbladder disease.

Gemini

Gastrointestinal symptoms such as nausea, vomiting, and diarrhoea are most common. These effects often improve over time.

Q6. Safety

ChatGPT

GLP-1 agonists are generally safe but contraindicated in certain populations, including those with medullary thyroid carcinoma or pancreatitis history.

Gemini

These medications are safe for many individuals but require careful evaluation of contraindications and monitoring during long-term use.

Q7. Weight loss

ChatGPT

Weight loss ranges from approximately 5–20% depending on the drug, dose, and duration of therapy.

Gemini

GLP-1 therapies typically produce 10–20% weight loss, with variability based on patient and treatment factors.

Q8. Onset of effect

ChatGPT

Glycaemic effects appear within weeks, while weight loss develops gradually over months.

Gemini

Initial effects occur within weeks, with full benefits developing over several months.

Q9. Discontinuation

ChatGPT

Stopping therapy often leads to weight regain due to reversal of appetite regulation mechanisms.

Gemini

Weight regain is common after discontinuation, highlighting the chronic nature of obesity.

Q10. Drug differences

ChatGPT

Ozempic, Wegovy, and Rybelsus all contain semaglutide but differ in indication, dosing, and administration route.

Gemini

These medications share the same active ingredient but differ in dosing, delivery method, and approved use.
