# Supplementary material for: Readability of AI-Generated Patient Information on Glucagon-Like Peptide-1 Receptor Agonists
Source: JMIR Bioinform Biotechnol. 2026 May 5;7:e90572. doi: 10.2196/90572 (PMC13143154; doi:10.2196/90572)
Supplement: Multimedia Appendix 3 [file bioinform-v7-e90572-s003.docx]

Supplementary Table S1

| **Metric** | **ChatGPT** | **Gemini** |
| --- | --- | --- |
| FRES (mean) | 31.65 | 47.97 |
| FKGL (mean) | 13.1 | 10.2 |
| Interpretation | Difficult | Moderate |
